# Evaluation of Recyclable Multilayer Packaging Designs Utilising Controlled Interlayer Adhesion

**DOI:** 10.1007/s11340-025-01200-2

**Published:** 2025-06-16

**Authors:** M.C. Mulakkal, C. Ekins, J. Wen, R. Ramchandran, A.C. Taylor, S. Pimenta, M.N. Charalambides

**Affiliations:** 1 Department of Mechanical Engineering, Imperial College London, South Kensington Campus, London, SW7 2 AZ UK; 2 Director Packaging R & D AMESA Foods Pepsico India Holdings Pvt Ltd, Gurgaon, India

**Keywords:** Recycling, Multilayered packaging materials, Plastic packaging, Localised adhesion, Water-soluble adhesive layer

## Abstract

**Background:**

The packaging industry is utilising increased levels of bio-based or recycled plastics and virgin plastic-based packaging is irreplaceable in more demanding applications such as food and pharmaceutical storage where different types of functional plastics are combined in a laminate form to produce multilayered packaging (MLP). Even though MLP are very effective in packaging applications, the typical multilayer format is a barrier to effective recycling, limiting the value and market for the use of recovered materials.

**Objective:**

This article investigates two new multilayer packaging design concepts which enable separation of the constituent layers in MLP. In these designs, the typical thermoset based adhesive layer in MLP is replaced by (i) localised adhesion by patterning surface treatments on the layers (no dedicated tie-layer) and (ii) by a water-soluble adhesive layer.

**Methods:**

T-peel testing is performed to evaluate the level of adhesion. The feasibility of these designs to enable layer separation was also investigated through representative tests that the simulated typical processes of shredding and washing in recycling streams.

**Results:**

The effectiveness of masks to localise surface treatment and thus create regions of higher and lower adhesion was captured in the peel test results for design A. The comparatively low levels of adhesion in design A enabled an easy separation of layers. An excellent adhesive was observed in peel test for design B with water soluble tie layer and the layers were separated by dissolving the tie layer in water.

**Conclusions:**

These concepts targeting the interface between MLP layers can be scaled with MLP complexity. Potentially, a combination of the two strategies could yield an optimal solution, where the total surface area of adhered MLP is reduced due to localised adhesion and a distinct water-soluble adhesive layer provides the necessary adhesive strength comparable to current MLP applications.

**Supplementary Information:**

The online version contains supplementary material available at 10.1007/s11340-025-01200-2.

## Introduction

Multilayer packaging (MLP) are widely used in packaging by industries such as food and pharmaceuticals to extend the shelf life for the products packaged within. These MLP are essentially layers of different materials (mostly plastics and metallic foils) stuck together in a laminate form. This type of construction allows to them meet demanding specifications such as mechanical support, barrier protection (against light, humidity, and oxygen), printability (for branding and communication) and sealability (to form closed packaging) with the lowest material consumption. The complexity of packaging (i.e. number of layers and materials used) are determined by the type of the packaged product and the service environment. Currently, MLP accounts for 26% of all flexible packaging and represents the largest portion of nonrecyclable packaging with end of life options limited to landfill or incineration for energy recovery [1, 2]. Contemporary recycling systems are geared towards recovering and recycling monomaterial items from mixed plastic waste, and the presence of MLP often results in contamination through incorrect sorting by means of near infrared (NIR) technology [3]. Collecting and recycling MLP on their own through melt blending techniques results in low mechanical properties and significantly limits the re-use potential [4–7]. The inability to separate the layers in MLP is a major limitation in utilising the existing recycling pathways for the constituent polymers. Although compatibilisation (melt blending with the addition of a chemical that enhances the interface between different polymers) has been shown to be able to deal with immiscible polymers in exclusively polymeric MLP waste [8–10], such melt processing options are inadequate to deal with flexible packaging that contains metallic foils and metal-coated polymeric films [5, 6]. There are options to recover the metal (whether laminated foil or metallised polymer) through the use of novel reagents or solvents [11], although the polymeric backing is often not recovered. Polyethylene and polypropylene based monomaterial packaging products have been developed which claim to meet the packaging requirements typically met by conventional MLPs. However, these typically lack in high barrier properties found in metallised laminates which offer better barrier protection than monomaterial packaging while utilising less materials. Besides the presence of metallised layers, there are factors such as inks, additives, inadequate waste management systems and littering that limit the recycling potential of MLP [12]. Therefore, new MLP designs that facilitate recycling without compromising performance and costs are essential.

MLP laminates are engineered to perform their packaging functions at the lowest possible cost and weight. Prioritising recycling during the packaging design stage can ensure low environmental impact of the technologies used. The environmental benefits of extended shelf-life from the product and overall weight reduction could outweigh the benefits from improved recyclability by direct substitution especially if the recyclable layer is of lower performance. Therefore, it is imperative to consider the overall environmental performance of the packaging [13]. There are well-established “design for recycling” guidelines for rigid plastics packaging and electronics equipment, some examples are listed in References [14–17]. Furthermore, design guidelines and tools covering packaging parameters such as material choices, colours, additives, closures, barriers and labels pertaining MLP are being developed by many stakeholders across the globe [18–22]. The key to recycling MLP is to design products such that they retain maximum value in recycling; enabling separation of layers, minimising colours and printing are central to this.

From a packaging design perspective, several approaches to enable layer separation can be found among patents and in the literature. A common approach is to incorporate an interlayer that can be removed to release the other layers from the laminate. The interlayer could be dissolved or expanded in a solvent, or softened on the application of heat, which is then followed by mechanical separation by means of shearing of the layers [23–26]. Typically, thermoset polyurethanes are used as a thin adhesive layer to combine the different functional layers through lamination or co-extrusion. Whilst selective dissolution–precipitation methods can be applied to sequentially separate the different layers in MLP, such form of physical recycling necessitates appropriate solvents for the target polymers in MLP. There are many solvent-based approaches that can target the adhesive layer in multilayer packaging. It must be noted that this approach is distinct from the dissolution–precipitation methods [27, 28], as the separated polymers are aimed to be recycled through mechanical means which is the prevalent form of recycling today. Saperatec GmbH reported a proprietary separating liquid which is a nano-dispersion of organic components and surfactant molecules where the organic component swells the adhesive between the layers and surfactants intercalate between the layers to aid separation when mechanically agitated [29, 30]. Mumladze et al. [31] reported the use of switchable hydrophilicity solvents (N,N-Dimethylcyclohexylamine (DMCHA)) to separate the layers in MLP [31]. Cinelli et al. [32] reported whey protein isolate based coatings as an oxygen and moisture barrier layer which could be degraded by enzymes to enable delamination in MLP [32]. Kaiser et al. reported the use of a reversibly crosslinkable polyurethane adhesive layer which could be dissolved in a solvent to delaminate the layers [33]. With regards to any solvent based delamination methods, life cycle analysis (LCA) studies play an important part in determining the impact of such technologies on the environment. An alternative approach is to limit the adhesion between the layers. Packaging laminate prototypes that are only adhered around the periphery have been described in patent EP3059061 A1; mechanical shredding releases the unstuck layers from the laminate and the multilayer components can be separated through a sink/float process [34]. Furthermore, recent developments have reported on thermoreversible polyurethane-based adhesives that allow reversible adhesion by means of a breakdown in adhesive structure in response to heat stimuli [35]. These adhesives when combined with a thermal absorber such as graphene allows for targeted heating of the adhesive layer and breakdown of adhesive structure in short timescales [36]. Even though these works have demonstrated its merits, the current MLP design remains largely unchanged. It can be noted that these approaches described here require a dedicated processing steps that target layer separation. There are benefits to developing new recycling approaches that pose minimum disruption and do not necessarily add additional steps in recycling stage to enable layer separation. This article describes such developments.

MLP laminates manufactured through lamination or extrusion-lamination processes typically undergo extensive surface treatments such as plasma or corona discharge to enhance the adhesion between the layers. Applying surface treatments localised in patterns across the film surfaces prior to lamination could be an interesting option to control the levels of adhesion between the layers. Shredding such laminates down to areas smaller than the imposed adhesive pattern releases the non-adhered layers. During recycling steps, plastics undergo shearing (when passed through rotating shredder teeth) and washing steps for size reduction and to eliminate contaminants. Our research attempts to leverage these inherent operations in the manufacturing and recycling stages to propose and evaluate two recyclable designs for MLP by means of (a) controlling the level of adhesion between the layers through localised surface treatments (oxygen plasma surface treatment), and (b) using a water-soluble inter-layer adhesive from Polyvinyl alcohol (PVOH). Although PVOH is used in packaging as a barrier film, its use as an adhesive layer and especially as a removable adhesive layer has not been previously reported. In this work, the materials, design and manufacturing of these recyclable MLP are reported followed by experimental characterisation of interlayer adhesion, durability and an evaluation of recyclability by verifying delamination of the laminates into separate layers. The adhesion strategies investigated in this paper are novel in the context of recyclable packaging; their characterisation is important towards enabling enable the recycling of hard to recycle multilayer flexible plastics.

## MLP Designs, Materials and Manufacturing

### Laminate Designs

**Fig. 1:**
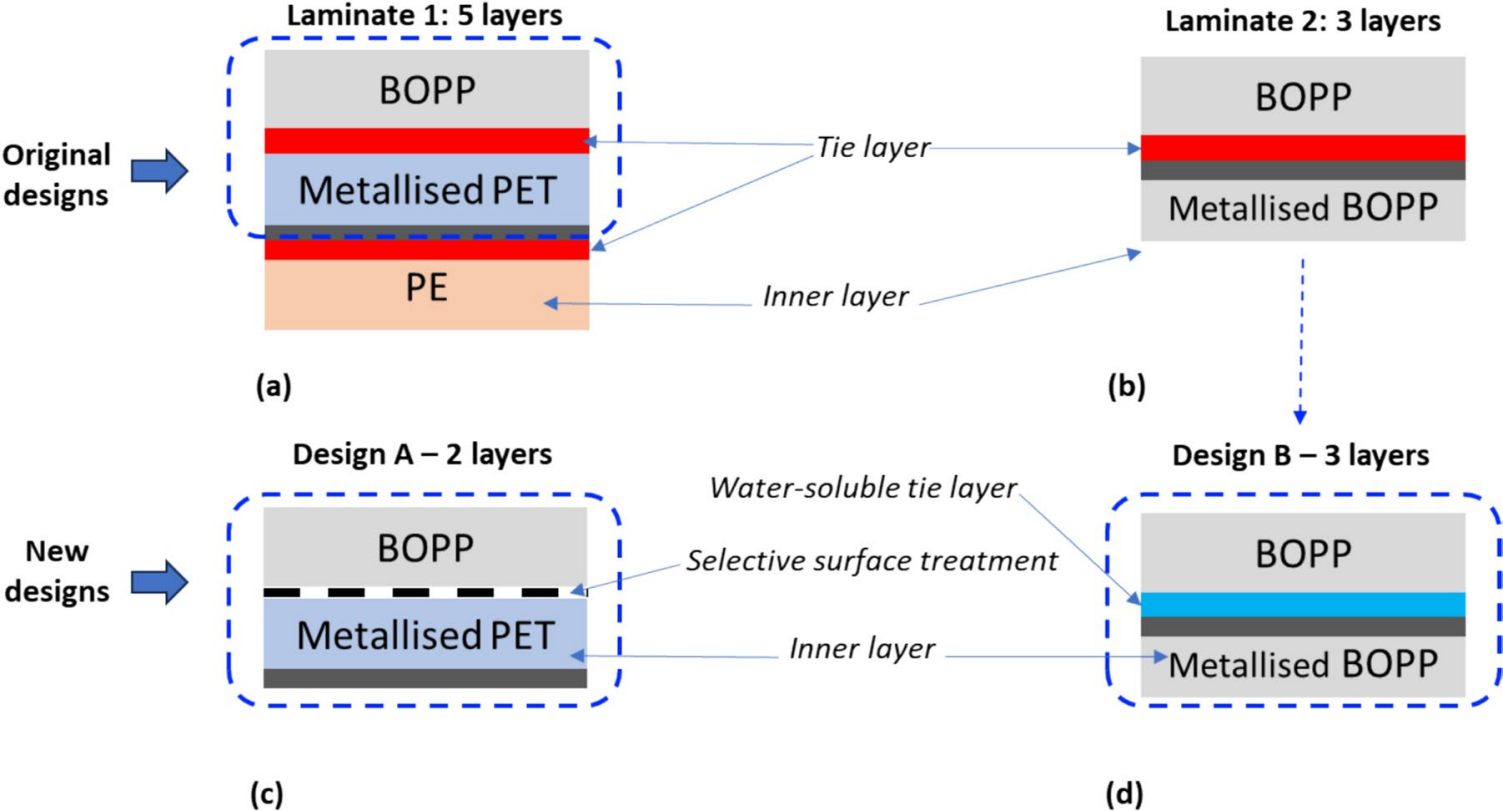
Original laminate designs of Laminate 1 (**a**) and Laminate 2 (**b**), and the simplified versions utilised in this study; Design A (**c**) and Design B (**d**). BOPP- biaxially oriented polypropylene, PET—polyethylene terephthalate

Two packaging laminates (identified as Laminates 1 and 2 in Fig. 1) that are used as packaging in the fast-moving consumer goods (FMCG) sector in Asian and European markets respectively were chosen for this study. The 5-layer construction of Laminate 1 is typical for snacks packaging for the tropical market which necessitates more stringent packaging requirements, while the 3-layer construction of Laminate 2 is typical for the less demanding environment of the European market. Metallised biaxially oriented polypropylene (MET BOPP) and metallised polyethylene terephthalate (MET PET) are typically produced using vapour deposition techniques, in which the metal in vapour form is condensed as a very thin metal layer onto the respective polymer layers; aluminium is usually used to metallise food packaging applications, and only one side of the polymer sheet (non-food contact) undergoes this treatment. When such layers are used as the innermost layer in a packaging, sealing of the packaging is typically achieved through hot sealing the polymeric side to itself. Traditionally, thermoset polyurethane adhesives are utilised as tie layers in MLP laminates.

In this study, two simplified laminate designs (A and B, shown in Fig. 1) derived from these existing MLP laminate configurations were considered, to evaluate the recycling potential of (A) localised surface treatments and (B) water-soluble tie layer (as alternatives to the traditional thermoset adhesives, which restrict recycling by limiting layer separation). This section details the principles used to design the modified Laminates A and B; more information on the materials utilised is given in Sect. 2.2, and the steps involved in the manufacturing process are detailed in Sect. 2.3.

As interlayer adhesion is the focus of this study, laminate architectures with 2-layer is the minimum required to evaluate the proposed adhesion strategies. Therefore, the 5-layer structure of Laminate 1 was simplified by removing the tie layers and innermost polyrthylene(PE) layer; this configuration is denoted as Design A and the adhesion here will be a function of surface treatment and level of material fusion at the interface as the tie layer is removed. In this study, surface treatment on the layer surfaces to be adhered will be localised through the application of a mask of specific pattern and compared to laminates where surface treatment was applied uniformly on the surfaces to be adhered (i.e. without a mask). It is likely these measures to create localised surface treatments could increase the production costs. However, this is an approach that could be applied in-line to existing manufacturing set-ups and could enable separation of MLP layers in typical recycling steps rather than targeted/additional layer separation steps. Interlayer adhesion between different polymers such as polypropylene (PP) and MET PET arising from material fusion alone will be lower. Ideally, the selectively patterned surface treatments should be applied in conjunction with a tie layer to ensure sufficient adhesion to withstand the requirements pertaining to adhesion and overall structural integrity during the filling and storage stages of the product packaging. Eliminating such a tie layer in our study for this Laminate 1 will enable us to decouple the influence of locally treated surface patterns from the functions of a tie layer.

**Fig. 2:**
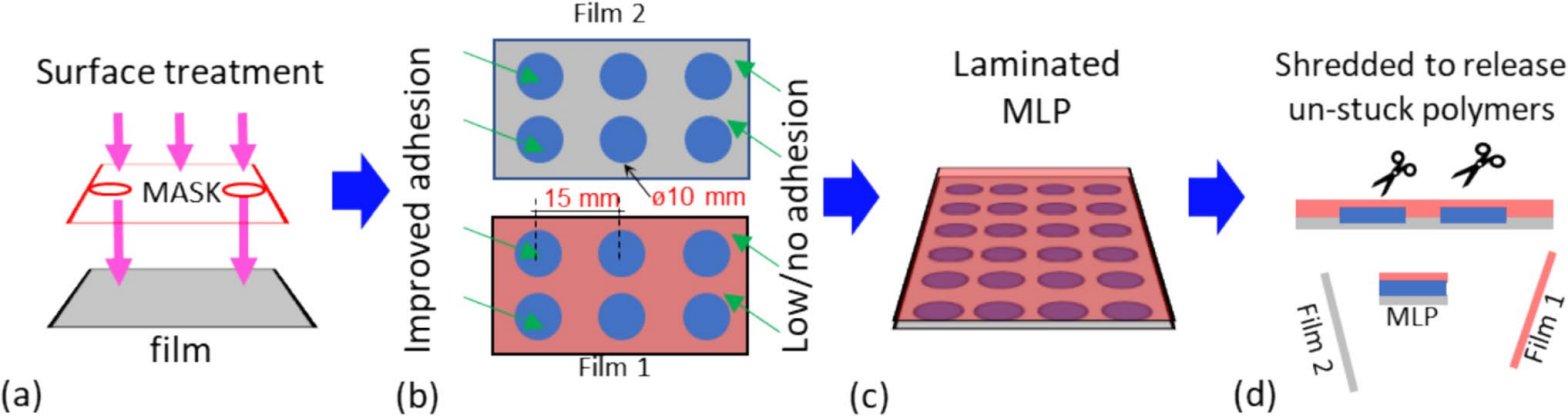
Schematics of localised adhesion design: (**a**) surface treatments, (**b**) regions of improved and low adhesion as a result of masked treatment, (**c**) manufacturing MLP through lamination, (**d**) recycling – shredding and washing to release unstuck polymer layers

Furthermore, it is essential to investigate the shredding mechanism of flexible MLP plastics in recycling facilities to devise the design of the pattern for localised surface treatment. The adhesive pattern design must be optimised such that the laminates are structurally comparable to the designs with uniform surface treatments whilst enabling separation during the recycling stages of shredding and washing. A selection of recycling facilities and shredding machine manufacturers were contacted to obtain information on their shredding processes. Typically, linear shredder spacing of 15 mm is commonly used to produce strips of flexible plastics [37]. Figure 2 shows a schematic of the steps involved in the manufacture and recycling of a locally adhered MLP. Figure 2(a) shows how regions of improved adhesion are generated on a surface through the surface masks. The specific details on the surface treatments are provided in Sect. 2.3. An initial pattern of circles with diameter 10 mm and spacing of 15 mm was chosen and applied on to the BOPP and PET side of MET PET, as shown in Fig. 2(b). Figure 2(c) shows a schematic of two films with localised surface treatments and laminated to manufacture a selectively adhered MLP. Figure 2(d) shows the separation of laminated MLP film by shredding into small pieces of MLP and unstuck polymer films. This concept of ‘Design A’ was chosen to validate our approach towards optimising the adhesion patterns without compromising packaging integrity.

As for Laminate 2, the polyurethane (PU) based thermoset tie layer is replaced with a water-soluble tie layer prepared from Mowiflex material in the modified Laminate Design B. In addition to shredding to reduce the size, collected plastic waste articles typically undergo several cleaning steps such as friction wash and hot wash prior to recycling. Therefore, utilising a water-soluble polymer as a tie layer in MLP (as in ‘Design B’) could aid in layer separation and subsequent recycling.

### Materials

The laminae, namely biaxially oriented polypropylene (BOPP), polyethylene (PE), metallised BOPP (MET BOPP) and metallised polyethylene terephthalate (MET PET), were supplied by PepsiCo. Besides the solubility in water, the adhesion, extrudability (shear thinning) and temperature stability are also important in selecting a tie layer material suitable for Design B. Therefore, a water-soluble polyvinyl alcohol (PVOH) based adhesive material [38] supplied by IMCD UK was selected. It is possible that the laminate could potentially be more susceptible to moisture absorption from surrounding environment and may result in premature delamination. However, this aspect could be managed through effective laminate design, for example by increasing the thickness of one of the outer layers or by utilising an improved barrier layer to limit moisture transport into the water soluble layer. Furthermore, PVOH is used in some packaging as a barrier against oxygen. Therefore, the choice of Mowiflex C17 material, which is based on PVOH resin, is justified to explore this concept. The particular grade used is formulated to function as a water-soluble support material in fused filament fabrication (FFF) additive manufacturing (3D-printing) processes. Table 1 shows the thickness of the polymeric films used to manufacture the laminates investigated in this study as well as the typical range of melt temperatures for these polymers. The latter is important as it dictates the lamination temperature. By virtue of the process involved in manufacturing oriented plastic films, there exists a directional bias namely machine direction (MD) and transverse direction (TD) that are orthogonal to each other. Machine direction lies along the direction of extrusion (material travel) in manufacturing. During the biaxial orientation step, the extruded material is then further stretched in MD and TD directions after extrusion. Mechanical properties such as modulus are different in these orthogonal directions mainly due to the different alignment of molecules as a result of this manufacturing process. These directions were not identified in the supplied materials but were determined through tensile testing of samples extracted from the supplied material as reported in Sect. 3.2.

**Table 1:** Materials and their properties

		Laminate 1			Laminate 2	
Property	Symbol	Design A			Design B	
		BOPP	MET PET	PVOH	BOPP	MET BOPP
Thickness (µm)	t	19.2 ± 0.2	12.9 ± 0.5	9.7 ± 2.3	23.1 ± 0.6	22.0 ± 0.7
Melting point (°C) [39]	T_m_	160–200	260–280	165–170	160–200	160–200

*PE*, polyethylene; *BOPP*, Biaxially oriented polypropylene; *MET*, metallised; *PET*, polyethylene terephthalate.

### Manufacturing of Laminates

#### Design A

Surface treatments are typically applied to the individual layers prior to lamination to improve adhesion. However, localisation of surface treatment to control adhesion is not common in the packaging industry even though it has been successfully applied in the fields of microelectronics and biomechanics for manufacturing precise circuit boards and microfluidic channels [40–42]. In the case of BOPP and MET PET, a substrate mask with a pattern of circles with a diameter of 10 mm and spacing of 15 mm was utilised to confine the surface treatments to the patterns as opposed to uniform application. The masks were made out of a 3-mm-thick plastic sheet of the same material as the film subjected to treatment. This was chosen to avoid any possible cross contamination of the surfaces due to ion deposition from a different mask material. The patterns were transferred from 2D CAD drawings onto the mask using a laser cutter. A pure oxygen plasma surface treatment was performed on the individual films utilising a Diener Pico barrel vacuum treatment system from Diener Electronic. The surfaces were treated at 90% power (270 W) for three minutes at ≈ 0.4 mbar pressure and constant flow rate of 5 ml/min. The films of BOPP and MET PET were cleaned with deionised water and thoroughly dried by wiping with lint free paper to ensure the surfaces were clean prior to plasma application.

Films were cut to 270 mm × 95 mm rectangles (dictated by the plasma chamber dimensions) to be plasma treated. The rectangles were cut such that the machine direction (MD) of the layers aligned with the long edge. The machine direction and transverse directions were determined by tensile tests as reported in Sect. 3.2. Alignment guides were engraved onto the mask substrate with a laser to ensure repeatable alignment of the pattern with the film surface prior to surface treatment. A multimeter was utilised to identify the metallised and non-metallised surfaces of MET PET film by measuring their electrical resistance. As per Design A, surface treatments were applied to the BOPP and the PET side of the MET PET film.

A dry film hot roll photoresist laminator (XRL 120) from Western Magnum was used to laminate the films. The films were stacked with the treated surfaces facing each other and were sandwiched between two 100 gsm A4-size printer paper sheets. These were then passed through the hot rollers at a temperature of 125 °C (for both top and bottom rollers) at a clamping pressure of 345 kPa (50 psi) and drive speed of 18 cm/min. The temperature was chosen to ensure sufficient adhesion without compromising the dimensional stability of the BOPP (see melt temperatures in Table 1) and also to minimise the subsequent curling of the laminated films. The laminated films of BOPP-MET PET bi-layers curled up into tubes after removal from the paper sleeves as a result of locked-in internal strains owing to their thermal expansion mismatch (see supplementary Figure S1). In addition to the difference in the coefficients of the thermal expansion between the polymers, it is possible that the BOPP layer shrank during cooling by an even greater amount due to heat-induced loss of axial orientation of polypropylene molecules compared to the MET-PET layer. The observed curling with the MET-PET on the outside and the BOPP on the inside of the tube confirms the greater shrinkage in BOPP.

#### Design B

Mowiflex C17 PVOH was supplied in extrudable pellet form but it was decided to use solvent casting to manufacture films as opposed to hot press as the former yielded thinner films (≈ 10 µm) that were more comparable to typical tie layer thickness found in MLP. The steps involved in film casting are as follows: 2.5 g of Mowiflex C17 was added to 50 ml deionised water and stirred on a hot plate with a magnetic stirrer. Once fully dissolved at room temperature (18 °C) forming a clear solution, the hot plate temperature was slowly raised to 100 °C to saturate the solution by removing excess water through evaporation. Once the solution neared the 25 ml mark, precipitation of the polymer started to occur as the clear solution started to turn cloudy. The prepared saturated solution was then covered with cling film and left overnight at room temperature to allow the bubbles formed during the mixing procedure to rise to the free surface. The optically-clear saturated PVOH solution was then poured onto a 100-µm-thick PTFE film and sandwiched by another 100 µm PTFE film. A silicone-coated hand-held roller was used to apply pressure on the sandwiched PTFE layers to facilitate uniform spreading of the PVOH solution. The sandwiched PTFE films were then manually separated and dried at room temperature to form PVOH films. Once solidified, these were carefully peeled from the substrate using a razor blade and tweezers. The PVOH film thickness was measured as 9.7 ± 2.3 µm using a digital micrometer from three films.

The PVOH film was sandwiched between BOPP and MET BOPP films (275 mm × 95 mm) with the metallised side in contact with the PVOH film as shown Fig. 1(d). The rectangles were cut such that the machine direction (MD) of the layers aligned with the long edge. The machine direction and transverse directions were determined by tensile tests as reported in Sect. 3.3 The BOPP—MET BOPP laminates remained flat unlike the BOPP—MET PET laminate of Design A, due to the same polymer (BOPP) used in both films of the Design B laminate.

## Experimental Procedures

### Water Contact Angle Tests

Water contact angle measurements were conducted on untreated and plasma-treated films to understand the efficacy of the plasma treatment used in the manufacture of the laminates and its localisation through the mask application. The angle formed by the outer edge of a water droplet to the substrate it is placed upon is indicative of the substrate’s wettability or surface energy. A small contact angle (less than 90˚) indicates high surface wettability and a greater level of adhesion compared to a higher contact angle (greater than 90˚). Typically, surface treatment techniques such as plasma result in a high surface energy and lowers the contact angle of the surfaces leading to improved adhesion.

Contact angle measurements were taken utilising a goniometer from Ramé-Hart (model no. 100–200 230) from two locations greater than 10 mm apart. The measurements were taken from two separate specimens for each of the sample types, with seven to twelve discrete locations across the surfaces measured for each sample. Each of these measurements were recorded using the goniometer software where the angle recorded is the average angle recorded over a period of 10 s after droplet dispensing. Contact angle measurements were taken from un-treated and uniformly plasma-treated (i.e. no mask/localisation) samples as well as from masked and unmasked regions of the sample subjected to localised plasma treatment.

### Tensile Tests

The tensile tests were conducted on individual layers of materials in laminate Design A (BOPP and MET-PET) and design B (BOPP and MET-BOPP) utilising an Instron single column test rig with a 1 kN load cell according to ASTM D882[43] to obtain the mechanical properties. A minimum of five replicate specimens in both machine (MD) and transverse directions (TD) were tested at the rate of 10 mm/min. As the samples are less than 1 mm in thickness, ASTM D882 [43] was deemed appropriate to determine the tensile properties of the films. The test specimens were cut to dimensions with a sharp razor blade on a cutting mat. The films were cut to a width of 10 mm and a gauge length of 100 mm was utilised for all the tests. The thickness of each sample was measured at three locations (ends and middle) and averaged (see Table 1). The flexible films were loaded onto the test grips set 100 mm apart ensuring no slack and minimal preload was applied when closing the grips. The displacements were obtained from grip separation from the Instron. Elastic modulus was calculated between a strain interval of 0.001–0.002, whereas yield strength was calculated at 0.02 strain.

### T-Peel Tests

**Fig. 3:**
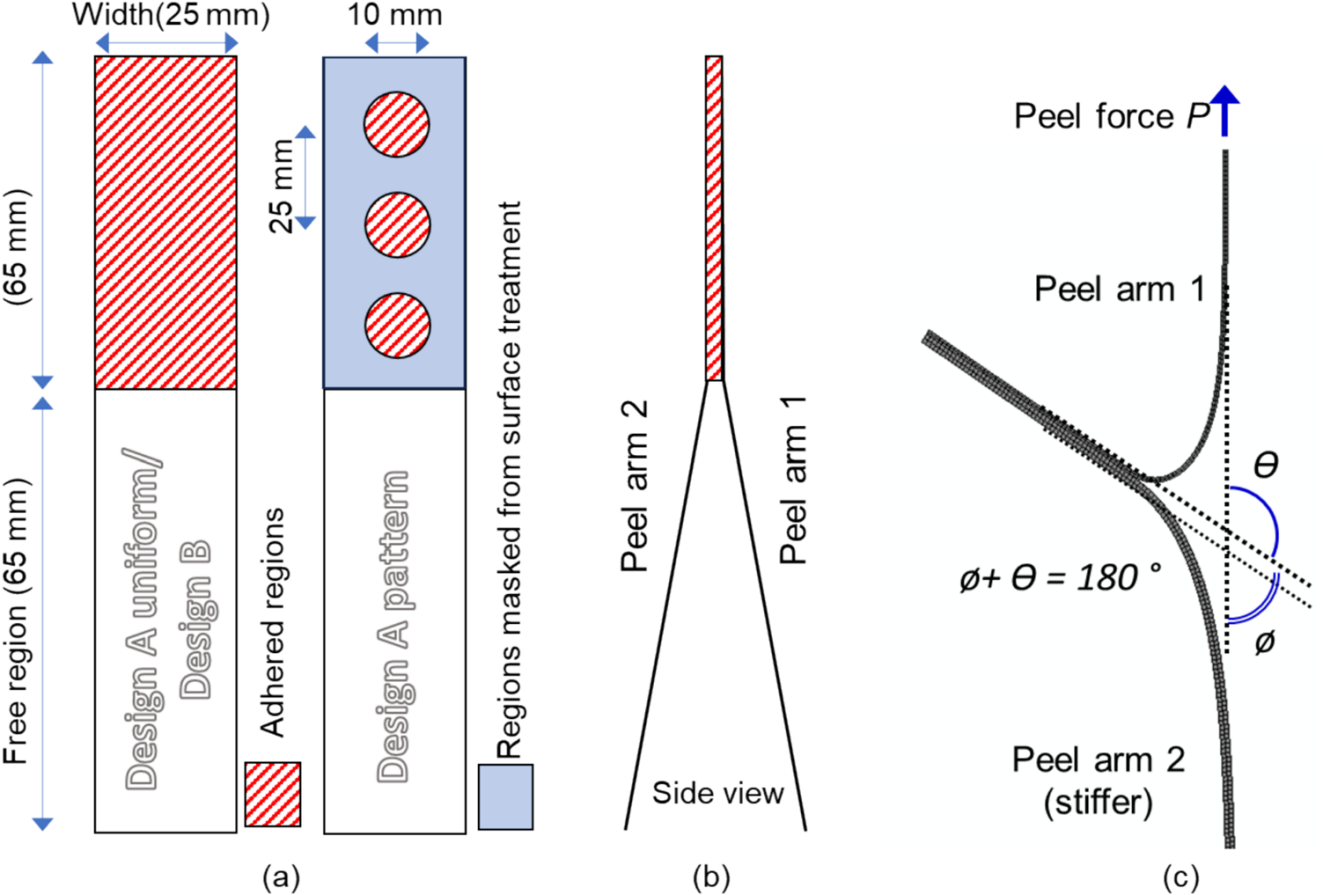
Schematics of T-peel test samples (**a**) Design A uniform, Design B and Design A pattern (**b**) T-peel test sample side view and (**c**) T-peel test set up

The adhesion between the laminates in Designs A and B was characterised by performing T-peel tests to determine the peel strength and adhesive fracture energy. An Instron single column universal testing machine with a 100 N load cell was utilised to conduct unsupported T-peel tests (as shown in Fig. 3) in accordance with ASTM F88/F88M-09 [44]. Four specimens were tested for each of the sample sets. Additionally, the peel angles for each of the peel arms ($\theta$ and $\phi$ in Fig. 3) were monitored and measured by means of a digital camera and image analysis software Fiji [45]. The peel angle is defined as the angle formed between the peel force and the substrate and was measured at the start, middle and towards the end of the peeling process and averaged for each specimen. For the example shown in Fig. 3, peel arm 1 has a peel angle of $\phi$ and peel arm 2 has an angle of $\theta$*.* These values are related as $\theta$ + $\phi$ = 180°. The peel strength (peel force per unit width) indicates how difficult it is to peel one substrate from another. However, peel strength alone is inadequate to characterise adhesion because it does not resolve the contribution of plastic bending during the peel process [46]. Therefore, the adhesive fracture energy needs to be determined as discussed in Sect. 4.3.2.

The test samples for Design A were prepared by carefully peeling back the fully laminated layers by hand up to halfway along the length (65 mm) to prepare free regions in the samples; and for Design B the layers were laminated such that the adhesive layer was only present for the adhered area with a paper insert to prevent bonding of the layers in the free regions during the lamination process (see Fig. 3). The specimens had a width of 25 mm for both laminate designs and the tests were performed at a speed of 200 mm/min.

### Evaluating Recyclability of New Laminate Designs

The two designs were evaluated for their recyclability within a laboratory setup that approximates the typical shredding and washing stages in the plastic waste recycling process. To this effect, laminates of both Design A and Design B were shredded. As the laminates of Design A (with patterned adhesion) resulted in curling (as shown in Supplementary Figure S1), it was decided to randomly cut the laminate tubes along the length and across the width by pressing down with a blade, then inspect the resulting shreds for delamination. However, more control could be achieved in the shredding of Design B as the laminates were not curled like Design A. The Design B laminates were then cut into 10 mm by 30 mm rectangle pieces (with a scalpel) and placed in a beaker containing deionised water. The pass/fail criteria for these tests would be to visually inspect whether the suggested steps result in the delamination of the layers in Design A and Design B as intended.

## Experimental Results

### Water Contact Angle Measurements

**Fig. 4:**
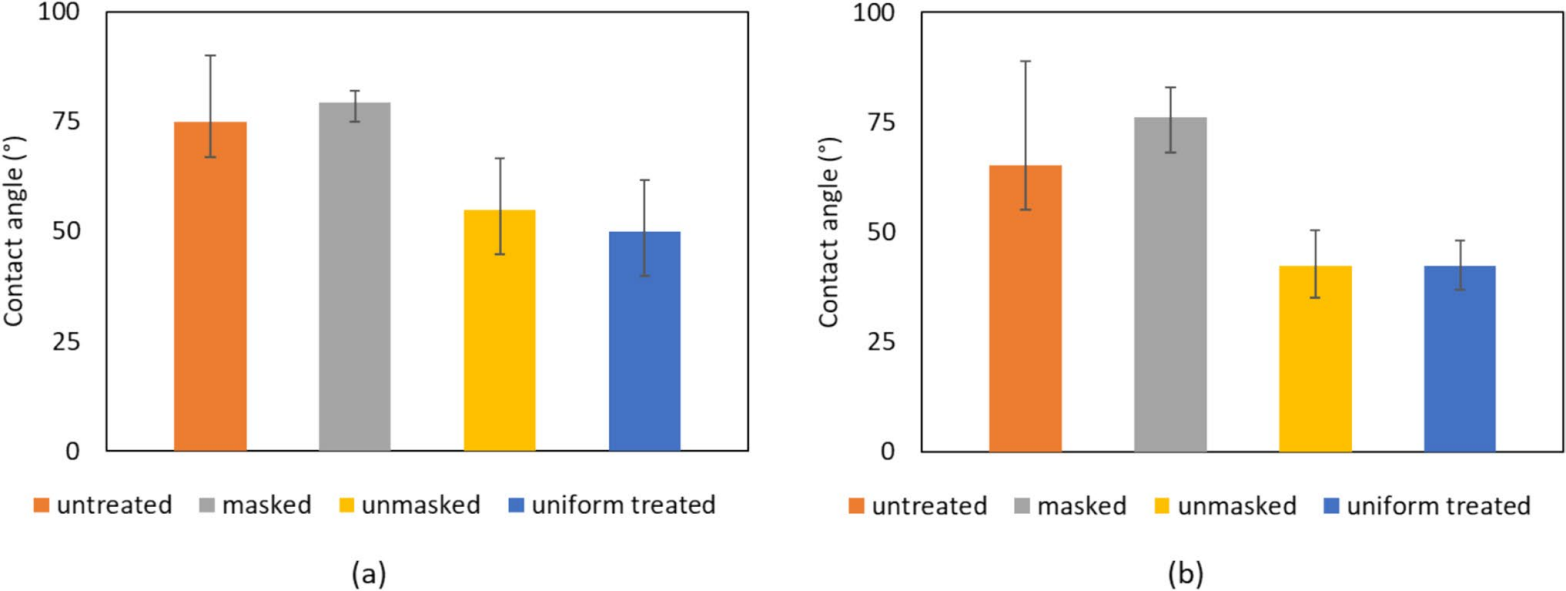
Contact angle measurements of (**a**) BOPP and (**b**) MET PET layers before and after oxygen plasma surface treatment. Error bars represent standard deviation

Figure 4 shows the contact angle measurements for samples before and after plasma treatment for BOPP and MET PET films. The untreated readings were taken from the films as supplied (i.e. without surface treatment and uniformly treated). The masked and unmasked readings were taken on samples subjected to plasma treatment with the mask applied. The results in Fig. 4 show that the application of masks resulted in successful localisation of the surface treatment effects and that the approach of localised masking result in contact angle of masked regions comparable to untreated specimens and unmasked regions comparable to that of conventional plasma treatment (uniform). The lower contact angles are indicative of regions capable of greater adhesion compared to regions with higher values within the film. The values shown are the average of 7–10 readings with the error bars representing standard deviation.

### Tensile Tests

Table 2 shows the average mechanical properties obtained from tensile testing with the error indicating standard deviation. A minimum of 3 to 5 specimens were tested for each direction.. As expected, the values are different in the two testing directions, and the MD direction was assumed to be that with lower modulus and yield stress, as is typically the case for biaxially oriented films. As for MET PET, the directional bias in properties is not as prominent as for the BOPP layers. It can be seen that the modulus (E) values for BOPP used in Design A and Design B have different values especially for MD. As the films are of different thickness, different draw ratios must have been applied during the biaxial stretching (orientation) process to obtain the set thickness. This could contribute to the difference in modulus and yield strength values between the two BOPP films. Furthermore, the treatment histories of the films (i.e. whether the layers were optimised for printing, adhesion to itself or laminating) could not be confirmed by the material supplier. As a result, comparison could only be made to the technical data sheets of the films with similar features and thicknesses. These test results are comparable to the data provided in technical datasheets for similar packaging films [47–50]. The Supplementary Figure S2 shows the stress–strain plots of (few selected specimens for clarity) all the laminates.

**Table 2:** Mechanical properties of the laminates

		Laminate 1		Laminate 2	
Property	Orientation	Design A		Design B	
		BOPP	MET PET	BOPP	MET BOPP
Modulus, E (MPa)	MD	1934 ± 85	4133 ± 171	1560 ± 100	1940 ± 76
	TD	3242 ± 443	4840 ± 382	3034 ± 170	4414 ± 404
Yield stress @0.02 (MPa)	MD	25.2 ± 1.6	88.4 ± 4.6	20.1 ± 0.4	27.4 ± 1.6
	TD	41.0 ± 1.7	89.8 ± 3.6	39.5 ± 1.3	61.3 ± 3.5

*PE*, polyethylene; *BOPP*, Biaxially oriented polypropylene; *MET*, metallised; *PET*, polyethylene terephthalate; *MD*, machine direction; *TD*, transverse direction.

### Adhesion

#### Peel force

**Fig. 5:**
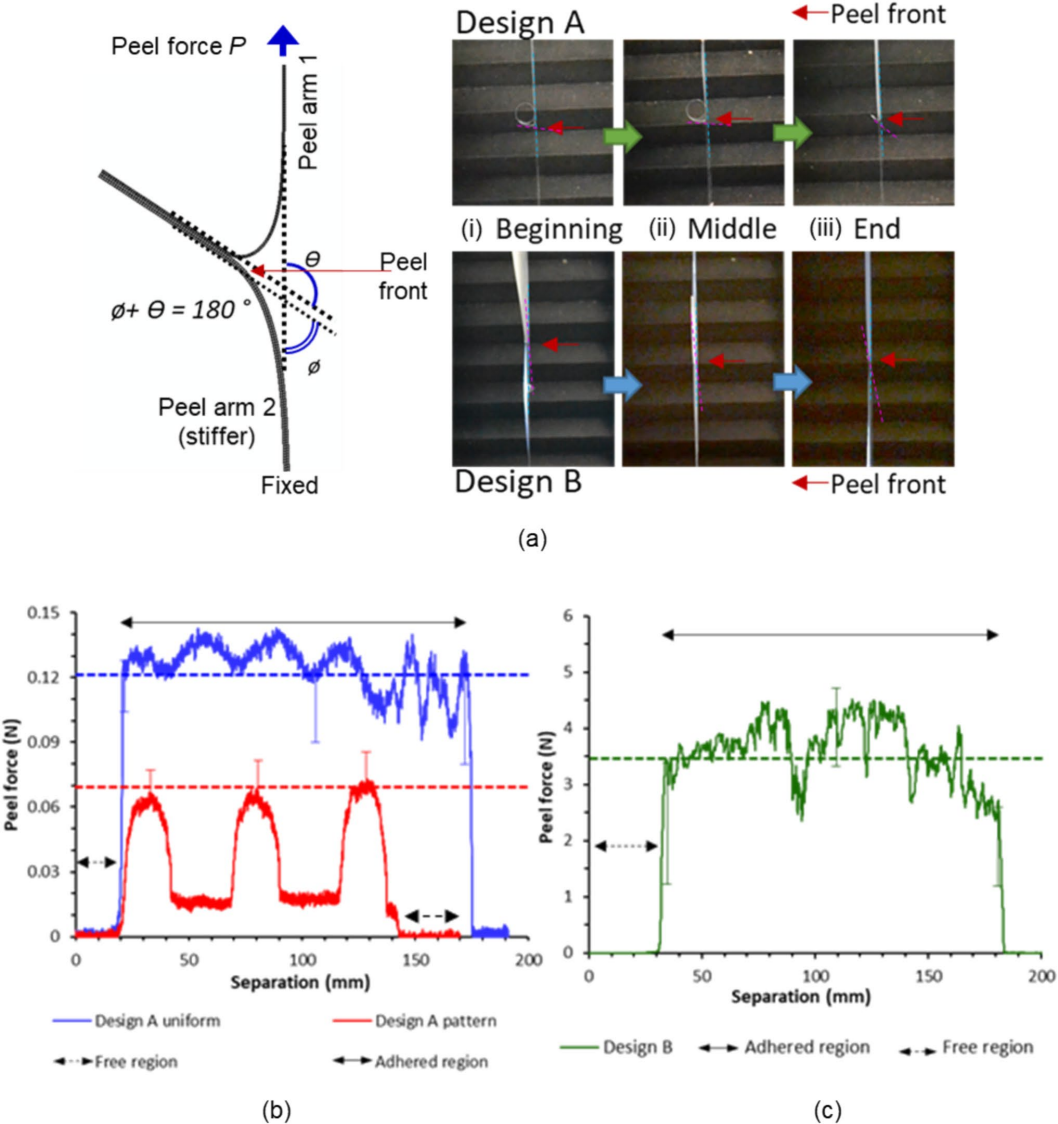
(**a**) Peel arm angle at the (i) beginning of peel (ii) middle and (iii) end of peeling process. Top row: Design A. Bottom row Design B. Peel force-separation distance plot for manufactured samples of (**b**) Design A and (**c**) Design B with a width of 25 mm

The specimen configuration in a T-peel test and the stages during peeling process are shown in Fig. 5(a). Peel force vs grip separation distance plots for a representative sample from each of the Designs A and B are shown in Fig. 5(b) and (c). The error bars on these plots demonstrate the spread of peel force at those specific separation distances (beginning of peel, middle and end) from 3–5 replicate test samples. The dotted lines represent the average peel force across 3–5 samples for each of the designs. The average peel force for Design A samples with localised adhesion (Design A pattern in Fig. 5(b)) was calculated by averaging the highest value in each of the peaks. For all other samples, the average peel force was calculated by averaging the peel force across the adhered region.

The average peel force obtained for uniformly surface treated and adhered samples of Design A is 0.120 ± 0.010 N. Using the sample width of 25 mm, this force corresponds to a peel strength of 0.005 N/mm. It must be noted that the value achieved here is much lower than the peel strength observed in commercial MLP films with a dedicated adhesive tie layer [0.13–0.28 N/mm]. For the samples with localised surface treatments, three distinct peaks corresponding to the three circular patterns were observed in all the tested specimens. The average value of these peaks across all the specimens was 0.070 ± 0.007 N. The average peel force in regions devoid of plasma treatment was 0.017 ± 0.007 N for all the specimens. It can be seen that the width of the peaks for Design A patterned samples Fig. 5(b) are 20 mm and corresponds to twice the diameter of the circle profiles that was patterned on the surface (as expected, since the rate of increase in separation should be twice that in peeled length, to account for the peeled length in both the top and bottom arms). The dome shape of the peaks are representative of the circle profile as the adhesion within these regions gradually increases as the peel front propagates through the pattern till it reaches the middle of each circle pattern and then decreases. The gap between the peaks (~ 30 mm) corresponds to twice the space between the patterned circle (also as expected). A clean adhesive failure was observed between BOPP and MET-PET with no apparent deformations of the peel arm post peeling (see Supplementary Figure S3a).

The average peel force of Design B specimens with the water-soluble PVOH adhesive was 3.45 ± 0.3 N (peel strength 0.14 N/mm). In most cases, failure of the seal was due to adhesive failure occurring between the top arm (BOPP) and the adhesive layer, with visible initiation of delamination occurring between the adhesive layer and the bottom arm (MET BOPP) only in a few samples. The peel force values did not change according to the location of the delamination. Plastic deformation or failure were not observed in the bottom MET BOPP peel arm nor the PVOH adhesive layer, however the upper peel arms (BOPP) were plastically deformed as they curled up into tubes along the length direction possibly due to the extensive plastic deformation of this peeled arm (see Supplementary Figure S3b).

#### Adhesive fracture energy

The fracture energy for the laminates in Designs A and B was calculated using the IC Peel Digitised algorithm [51] with the parameters derived from the tensile and T peel tests (see Sects. 5.1 and 5.2.1). The IC Peel algorithm is based on an analytical peeling model based on an energy balance and large displacement beam theory in order to calculate the fracture energy (also known as fracture toughness or interfacial work of fracture) [46] which was then implemented by Georgiou et al. [52] into an algorithm known as ‘IC Peel’. The latest version of this algorithm IC Peel Digitised (2007) [51] was utilised to calculate the adhesive fracture energy. The reader is directed to the references above for a detailed description of the peel analysis; however, a brief summary is given below.

During the peeling of two flexible laminates, the peel arms undergo a complex bending and unbending process and the input energy is resolved into deformational energies, elastic, plastic and adhesive fracture energies as follows.

1 $${G}_{a}=\frac{1}{b}\left(\frac{{dU}_{ext}}{da}-\frac{{dU}_{s}}{da}-\frac{{dU}_{t}}{da}-\frac{{dU}_{p}}{da}\right)$$

where *G*_*a*_ is the adhesive fracture energy, *dU*_*ext*_ is the external work, *dU*_*s*_ is the stored strain energy, *dU*_*t*_ the energy dissipated during tensile deformation and *dU*_*p*_ is the energy dissipated during plastic bending of the peel arm, whereas *b* is the specimen width and *da* is the peel crack length. It must be noted that the *G* terms have a unit of J/m^2^ and are essentially energy release rates.

For a T-peel test geometry of two peel arms, the total adhesive fracture energy *G*_*a*_ is the sum of the adhesive fracture energies of both peel arms (*G*_*a1*_ + *G*_*a2*_). The elastic and plastic deformation energies can be calculated from the stress–strain characteristics of the peel arm which include the initial elastic deformation as well as subsequent work hardening. Georgiou et al. [52] employed numerical and analytical modelling based on modified large-displacement beam theory to calculate the plastic bending in peel tests. These calculations can be performed with the aid of the ICPeel [51] software which was employed in this study. Based on this analysis, one can calculate the adhesive energy release rate from experimental stress–strain data of the peel arms (see Supplementary Figure S4), steady state peel load, peel angle and the sample dimensions. The peel angles were determined through image analysis using Fiji based on the video frames extracted at the beginning, middle and end of the peel process as shown in Fig. 5(a). An average of the peel angle at these three instances was utilised in the calculations. These parameters and the fracture energy values obtained from the algorithm are summarised in Table 3.

**Table 3:** Input and output parameters used in the IC Peel software for the fracture energy calculations

	Design A—uniform		Design B	
IC Peel (digitised) input	BOPP	MET PET	BOPP	MET BOPP
Peel force (N)	0.12	0.12	3.45	3.45
Peel angle (°)	73	107	179	1
Thickness of peel arm (µm)	20	13	23	22
Specimen width (mm)	25	25	25	25
Modulus of arm material (MPa) *(from single specimen)*	1875	4063	1597	1924
Yield point of arm material (MPa) *(from single specimen)*	26	87	20	26
IC Peel (digitised) outputs				
Input energy, G (J/m^2^)	3.40	6.20	276.2	0.25
Plastic bending energy, G_p_ (J/m^2^)	1.11	0.53	232.6	0
Adhesive fracture energy, G_a_ (J/m^2^)	2.28	5.67	44.65	0.25
Total fracture energy (J/m^2^)	**7.95**		**44.9**	

The MD properties of the peel arms were utilised in ICPeel analysis as the peel arm length coincides with this direction. However, due to the difference in MD and TD directions for mechanical properties (see Table 1), the calculated fracture energy should be considered as the best available approximation. It must also be noted that the values in Table 1 are sample averages while, IC Peel input require a single load–displacement curve and therefore the modulus and yield stress data shown in Table 3 are those of a single specimen than the sample average. The specimen whose modulus and yield point closer to the sample average was chosen for the single load-displacement curve into IC peel. The calculated values of adhesive fracture energy for Designs A and B can be compared to other laminate combinations in the literature. As with this study following the theoretical model of peel of Kinloch et al. [46], Islam et. al. reported an adhesive fracture energy of 33 J/m^2^ for a laminate of 25µm LDPE and 50µm PET [53]. Similarly, a value of 60–70 J/m^2^ was reported for a laminate of 6.3 µm thick Al foil and LDPE [54]. Assuming these are the typical adhesive fracture energy required of packaging laminates, only Design B falls within this range. As concluded from the peel tests, the adhesion in Design A is not sufficient to meet the requirements of packaging but a different strategy or increased surface treatment could yield improved results.

### Recyclability of New Laminate Designs

Design A samples, which were randomly sliced into smaller pieces by pressing a blade could be easily separated by manually shearing between fingers. This was the case for both surface treated and non-treated (masked) sections of the laminate. This meant that the recyclability of the design A could not be evaluated adequately. However, this is the result of not having an adhesive layer, resulting in lower adhesion between the layers in general (even in the unpatterned samples). Nevertheless, the localisation of surface treatments to enable lower adhesion between the layers in MLP has been successfully demonstrated in the T peel tests.

For Design B samples shredded and immersed in de-ionised water, the layers could be separated by manual shearing after overnight (≈12 h) immersion, with onset of partial delamination observed (without external influence of touching or shearing) after 24 h, and complete separation occurring after 36 h. It is possible to reduce this duration by introducing shear forces through agitation or by adding surfactant to the water to enable faster impregnation of water. Shredding the laminates into smaller pieces will also increase the surface area of the adhesive layer in contact with the water and speed up the process. Therefore, it has been demonstrated that the use of a water-soluble adhesive layer in MLP manufacturing can be a potent strategy to enable layer separation to facilitate MLP recycling.

## Discussion and Conclusion

The main aim for both Designs A and B was to demonstrate distinct routes for separating the layers in multilayer packaging (MLP). The peel strength of Design A was understandably low when compared to the values typically found in MLP due to the absence of a dedicated tie layer. The material fusion between BOPP and MET PET alone was not sufficient to reach the peel strength values reported for packaging MLP. However, the effectiveness of masks to localise surface treatment and thus create regions of higher and lower adhesion was captured (as shown by the peel test results in Fig. 5 (b)). The Design A laminates could be easily separated by shearing between the fingers, even before shredding them. Therefore it is recommended that Design A (pattern) either the strength of surface treatments to be increased or the patterns be employed with a dedicated tie layer in the future. In Design B, the water-soluble tie layer provided a level of adhesion comparable to MLP seen in packaging. This packaging could also be separated by dissolving away the tie layer after shredding into smaller pieces. However, the times noted for completely dissolving the tie layer can be impractical in a recycling plant where continuous flow of material is required to maximise yields. It must be noted that typically some form of mechanical agitation and surfactants are present in the cleaning stages of recycling and both can significantly reduce the time taken to fully dissolve the tie layer.

The presented designs were a simplified two-layer laminate that is comparable to MLP for FMCG in less demanding environments like Europe and is significantly different to the MLP required more stringent packaging requirements. The concepts investigated here are targeting the interface between MLP layers and can be scaled with MLP complexity. Potentially, a combination of the two strategies, namely localised surface treatment and a water-soluble tie layer could yield an optimal solution, where the amount of adhered MLP is reduced due to localised adhesion and a distinct water-soluble adhesive layer provides the necessary adhesive strength comparable to current MLP applications. This approach and its merits will be investigated in the future. In addition, the natural progression of the work presented here would be to characterise the barrier properties of these new laminate designs. The application of surface treatment to the layers is integral to current manufacturing processes and polymers such as PVOH are also used in packaging applications. Consequently, this concept does not warrant fundamental changes to the current and established manufacturing process for MLP. This is attractive for packaging manufacturers and thus fundamental to bringing recyclability by design to MLP packaging.

## Supplementary Information

Below is the link to the electronic supplementary material.

Supplementary file1 (DOCX 2844 KB)
