# Supplementary material for: Evaluation of Recyclable Multilayer Packaging Designs Utilising Controlled Interlayer Adhesion
Source: Exp Mech. 2025 Jun 16;65(8):1199–212. doi: 10.1007/s11340-025-01200-2 (PMC12446127; doi:10.1007/s11340-025-01200-2)
Supplement: Supplementary file 1 — Supplementary file1 (DOCX 2844 KB) [file 11340_2025_1200_MOESM1_ESM.docx]

**Supplementary figures**


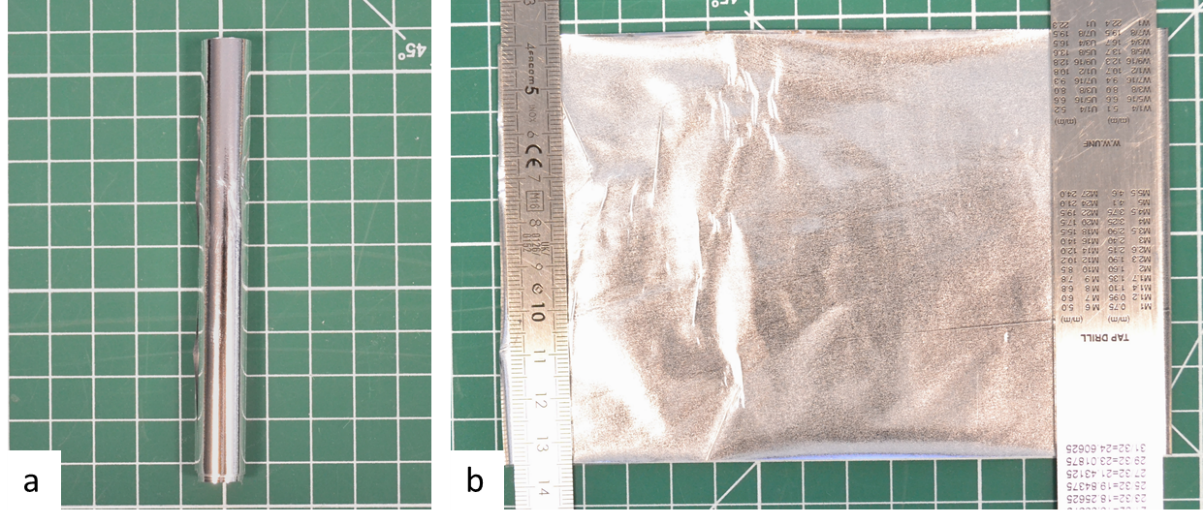


*Figure S1: (a) BOPP-MET PET laminate curled to form a tube after lamination (b) BOPP-MET PET laminate tube opened up and secured with the aid of weights. (Scale of square grid: 10 mm width)*

Figure S2: Stress - Strain plot of selected specimens for all the materials showing the variation across the specimens


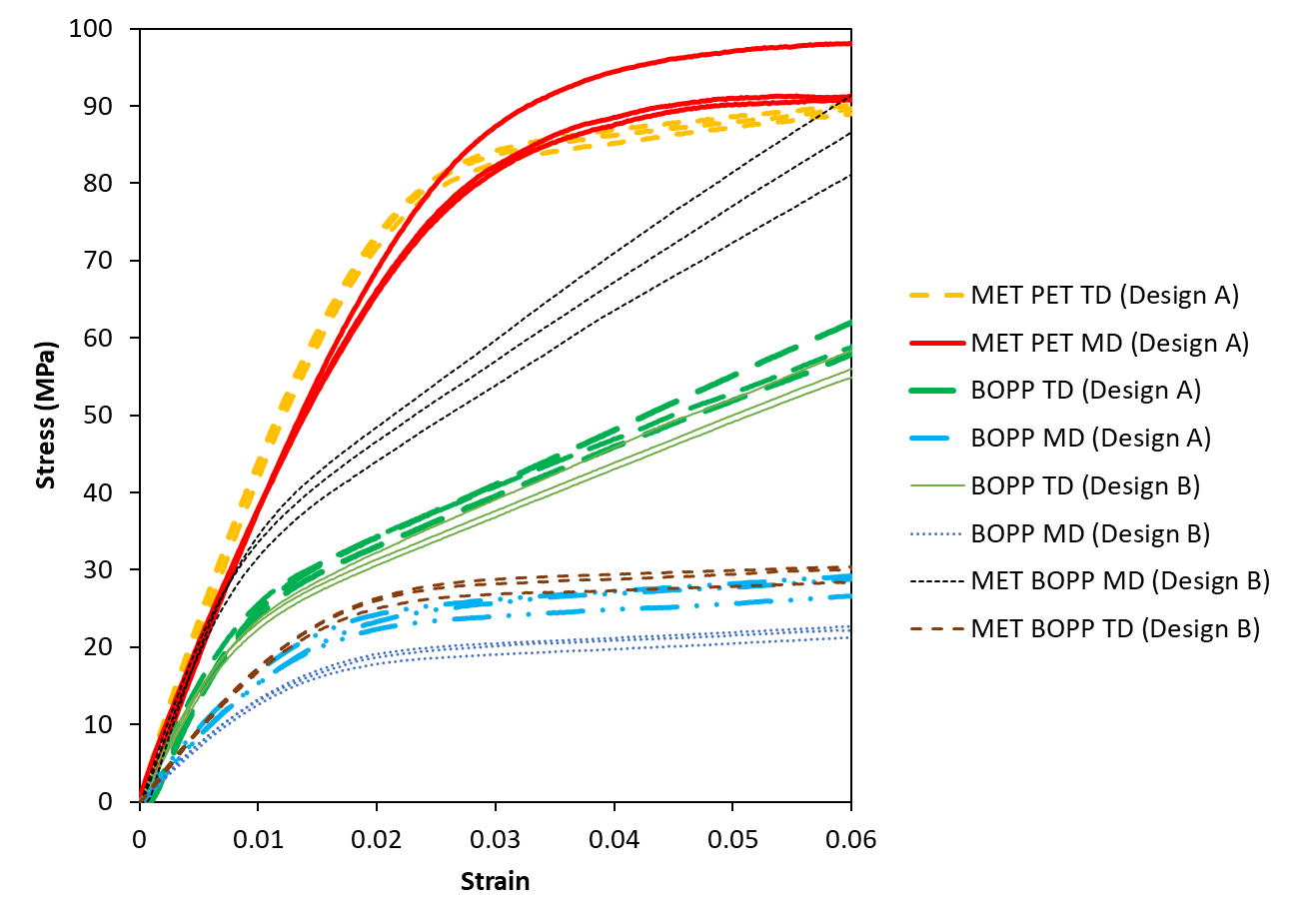


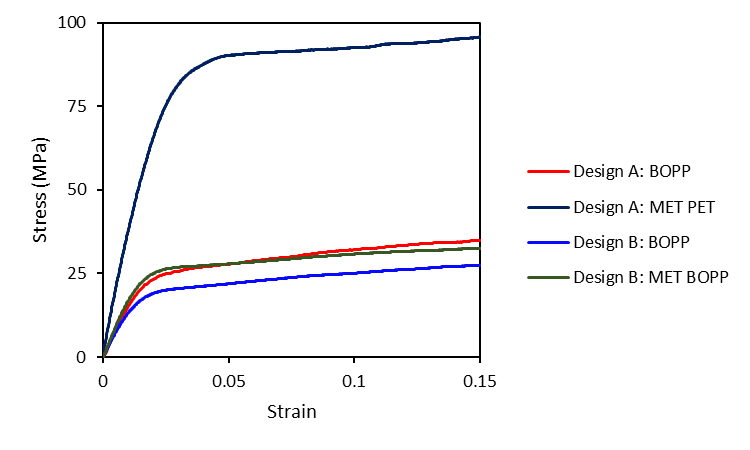

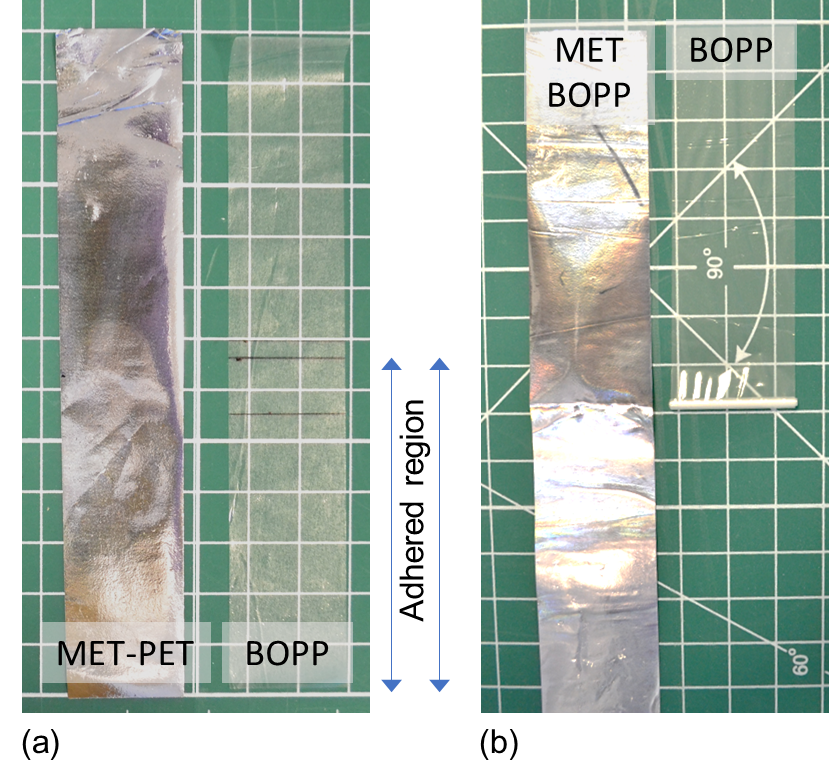


Figure S4: Experimental stress-strain data from a single specimen of peel arm materials in machine direction (MD) as used as input for ICPeel (Digitised).

Figure S3: Samples after T-peel test (a) Design A (pattern) and (b) Design B (Scale: 10 mm)
